# Sternoclavicular Septic Arthritis Caused by *Streptococcus pyogenes* in a Child

**DOI:** 10.5811/cpcem.2017.5.33811

**Published:** 2017-10-03

**Authors:** Yenisleidy Paez-Perez, Terrance McGovern, Ashley Flannery, Farid Naim

**Affiliations:** Department of Emergency Medicine, St. Joseph’s Regional Medical Center, Paterson, New Jersey

## Abstract

Septic arthritis can be a devastating condition that leads to further morbidity and potential mortality if not identified early in its course. Emergency providers must keep septic arthritis high on their differential of any joint-related pain in the pediatric population. We present a case of an eight-year-old female who initially presented with the chief complaint of chest pain and was subsequently diagnosed with septic arthritis of the left sternoclavicular joint in the emergency department.

## INTRODUCTION

Septic arthritis of the sternoclavicular joint is a rare entity, accounting for approximately 1% of cases in the general population and is most commonly caused by *Staphylococcus aureus.*^1^ Delayed diagnosis and management of septic arthritis can lead to subsequent deleterious complications such as osteomyelitis, local abscess formation, mediastinitis and poor functional outcomes.^2^ The superficial location of the sternoclavicular joint allows for recognition of obviously apparent edema early during the course of the disease process; however, the rare occurrence of septic arthritis in the sternoclavicular joint in healthy adults and presumed pediatric age group makes the diagnosis rather elusive.^1^

## CASE REPORT

An eight-year-old immunocompetent female with no past medical history presented to the emergency department (ED) with a two-day history of chest pain following a recent pharyngitis one week prior. Per the patient’s mother, 48 hours prior she began to complain of anterior and superior chest wall pain followed by a fever of 103°F with erythema and swelling over the left sternoclavicular joint, which became apparent during the preceding 24 hours. There was a substantial amount of erythema and tenderness over the left sternoclavicular joint, but no fluctuance was noted. There was also pain with active and passive range of motion of the left shoulder, especially with abduction and flexion of the joint. Radiographs showed no abnormalities including joint space widening, while the only laboratory abnormalities were a white blood cell count 23.5 K/mm3; C-reactive protein 38.9 mg/L; and erythrocyte sedimentation rate 44 mm/hr. Magnetic resonance imaging (MRI) of the chest was obtained in the ED to evaluate for possible septic arthritis, which revealed a marrow signal abnormality in the left medial clavicle and the sternum with an adjacent two centimeters fluid collection consistent with septic arthritis with an abscess. Blood cultures were obtained and the patient was started on clindamycin in the ED. Orthopedic surgery was consulted and patient was taken to the operating room for irrigation and debridement of the joint. Wound cultures grew *Streptococcus pyogenes* with no growth within the blood cultures. The patient was discharged from the hospital three days later with a peripherally inserted central catheter for continuous intravenous (IV) antibiotics for an additional four weeks. The patient has since made a full recovery.

## DISCUSSION

Septic arthritis of the sternoclavicular joint is an unusual occurrence in the immunocompetent population, accounting for 1% of septic arthritis cases and increasing precipitously to 17% in IV drug abusers.^1^,^3^,^4^ Additional risk factors for development of septic arthritis in the sternoclavicular joint besides IV drug abuse include diabetes mellitus, trauma, immunosuppression, renal failure, liver cirrhosis, distant site of infection or an infected central line, none of which our patient presented with.^1^ Current opinion holds that sternoclavicular joint septic arthritis is likely a result of hematogenous spread from a distant source or contiguous spread from a nearby infection into the sternoclavicular joint.^5^ Similar to the only previously reported occurrence of *S. pyogenes* sternoclavicular monoarthritis, we theorize that our patient developed septic arthritis of the sternoclavicular joint as a result of contiguous spread of the *S. pyogenes* from a previous pharyngitis acquired one week prior to presentation.

Sternoclavicular septic arthritis can be a difficult diagnosis to make in the ED, especially within the pediatric age group where there is a paucity of reported cases. Within the adult literature, insidious onset of chest pain localizing the sternoclavicular joint with associated redness and swelling is a common complaint occurring in 78% of cases.^1^ The presence of leukocytosis is only reported in 26–56% of patients who typically present to the ED over the course of two weeks since the onset of symptoms.^1^,^2^,^6^ Within the ED plain radiographs are typically of low yield, with computed tomography (CT) offering an increased sensitivity of 93% in detecting bony and soft tissue changes compared to radiograph.^7^,^8^ CT pales in comparison to MRI, which according to Kendrick et. al. has a sensitivity approaching 100% in diagnosing sternoclavicular joint septic arthritis.^9^

Obtaining a surgical specimen from the joint itself is the best method to confirm septic arthritis of the sternoclavicular joint. *S. aureus* is the most common etiologic entity appearing in about half of the cases, with *Pseudomonas aeruginosa, Brucella* and *Escherichia coli* as the other common causes, in decreasing order.^7^,^10^,^11^ In our review of the literature, we found numerous reported cases of *S. pyogenes* causing sternoclavicular septic arthritis in an immunocompromised adult patient or IV drug abusers; however, there has been only one reported case of in an immunocompetent adult patient.^1^,^12^,^13^

Further management of sternoclavicular septic arthritis includes initiating empiric parenteral antibacterial therapy with coverage against methicillin resistant *S. aureus (MRSA) and s. pyogenes* and continued for 4–6 weeks*.* Clindamycin or vancomycin are adequate first line *anti-staphylococcal* agents in an otherwise-healthy patient.^13^ In patients with immunosuppression or concurrent peripheral infection, antibiotics that target Gram-negative bacteria should also be included.

## CONCLUSION

To the best of our knowledge, we present the first pediatric case of sternoclavicular septic arthritis caused by *S.pyogenes* in an immunocompetent child who had no risk factors for this rare clinical entity. Septic arthritis must be considered in patients presenting with erythema overlying the joint, painful range of motion and fever. The exceedingly rare occurrence of this diagnosis makes it elusive in the ED, but the grave consequences of delayed diagnosis should raise the suspicion of any physician caring for children.

**CPC-EM Capsule**

What do we already know about this clinical entity?

Sternoclavicular joint septic arthritis (SCJSA) is a rare clinical entity that usually affects immunocompromised patients with contiguous or distant foci of infection.

What makes this presentation of disease reportable?

We present the first reported pediatric case of SCJSA caused by Streptococcus pyogenes in an immunocompetent child.

What is the major learning point?

SCJ infection may present with an insidious onset and the diagnosis may be missed, especially in a patient without predisposing risk factors.

How might this improve emergency medicine practice?

Physicians should maintain a high index of suspicion for SCJSA in patients presenting with atraumatic, painful swelling of the sternoclavicular joint, despite the lack of risk factors.
